# Supplementary material for: Effectiveness of the BNT162b2 mRNA Vaccine Compared with Hybrid Immunity in Populations Prioritized and Non-Prioritized for COVID-19 Vaccination in 2021–2022: A Naturalistic Case-Control Study in Sweden
Source: Vaccines (Basel). 2022 Aug 7;10(8):1273. doi: 10.3390/vaccines10081273 (PMC9414381; doi:10.3390/vaccines10081273)
Supplement: Supplementary file 1 [file vaccines-10-01273-s001.zip › vaccines-1833470-supplementary.pdf]

Supplementary Table S1. Total vaccinated population by 31 July 2021. Age and sex for the population vaccinated with the BNT162b2 vaccine and the proportions for the corresponding faction of the total adult population in Östergötland, Jönköping, and Skåne counties, Sweden (*n* = 576,526).

Age	Women			Men			Total		
	Vaccinated		All adults	Vaccinated		All adults	Vaccinated		All adults
	n	%	%	*n*	%	%	*n*	%	%
18–39 years	31,188	5	2	18,504	3	1	49,692	9	3
Prioritized	14,812	3	1	6865	1	0	21,677	4	1
Non-prioritized	16,376	3	1	11,639	2	1	28,015	5	2
40–64 years	124,679	22	7	121,930	21	7	246,609	43	14
Prioritized	54,147	9	3	38,607	7	2	92,754	16	5
Non-prioritized	70,532	12	4	83,323	14	5	153,855	27	9
65–79 years	97,870	17	6	89,739	16	5	187,609	33	11
Prioritized	90,473	16	5	82,886	14	5	173,359	30	10
Non-prioritized	7397	1	0	6853	1	0	14,250	2	1
80+ years	54,433	9	3	38,183	7	2	92,616	16	5
Prioritized	56,639	9	3	37,737	7	2	91,376	16	5
Non-prioritized	794	0	0	446	0	0	1240	0	0
Total	308,170	53	18	268,356	47	15	576,526	100	33
Prioritized	213,071	37	12	166,095	29	9	379,166	66	22
Non-prioritized	95,099	16	5	102,261	18	6	197,360	34	11
